# High variability in the assessment of physical function following total hip arthroplasty: a systematic review

**DOI:** 10.1097/JS9.0000000000003141

**Published:** 2025-08-26

**Authors:** Jonathan Lettner, Mona Ostermann, Nikolai Ramadanov, Claudia Sasse, Roland Becker, Robert Prill

**Affiliations:** a Center of Orthopaedics and Traumatology, University Hospital Brandenburg/Havel, Germany; b Faculty of Health Science, Brandenburg Medical School, Brandenburg an der Havel, Germany

**Keywords:** iBOMs, pBOMs, physical function, pROMs, systematic review, total hip arthroplasty

## Abstract

**Introduction:**

Total hip arthroplasty (THA) is a highly successful surgical intervention, yet the assessment of postoperative physical function lacks standardization. This systematic review investigates the variability in outcome measures used to evaluate physical function following THA over the past 12 years.

**Methods:**

Following PRISMA guidelines, we analyzed 406 randomized controlled trials (RCTs) from PubMed, Ovid, ScienceDirect, and Epistemonikos, focusing on studies published between 2013 and February 2025. Outcome measures were categorized into patient-reported (PROMs), performance-based (PBOMs), and impairment-based (IBOMs) measurement tools. Data extraction included study design, measurement types, and temporal trends.

**Results:**

Significant heterogeneity was observed, with PROMs employed in 95% of studies (e.g., Harris Hip Score, WOMAC), while PBOMs (18%) and IBOMs (22%) were underutilized. Only 8% of studies combined all three measurement types. PROMs dominated surgical studies (97%), whereas PBOMs and IBOMs were more prevalent in rehabilitation research. Temporal analysis revealed stable PROM usage but declining PBOM and inconsistent IBOM adoption.

**Conclusion:**

The lack of standardized functional assessment complicates cross-study comparisons and meta-analyses. While PROMs provide valuable patient perspectives, their limitations necessitate complementary objective measures. We advocate for the adoption of core outcome sets, such as those proposed by OMERACT, to improve consistency in THA outcome evaluation. Future research should integrate PROMs, PBOMs, and IBOMs for a comprehensive assessment of postoperative function.

## Introduction

Total hip arthroplasty (THA) is considered one of the most effective and successful surgical procedures in orthopedic medicine^[1]^. With approximately 250 000 procedures performed in 2022, THA ranks among the most frequently conducted orthopedic and endoprosthetic surgeries in Germany alone^[2]^. Given the increasing average age of the population, it is expected that these numbers will continue to rise in the coming decades^[3]^.

The necessity for THA can arise from various causes. One of the most common indications is hip osteoarthritis (OA), which leads to pain and functional impairments in the hip joint, significantly affecting patients’ quality of life. When conservative treatments are no longer effective in advanced hip OA, THA is considered the treatment of choice^[4,5]^. Other indications for THA include femoral neck fractures, aseptic necrosis of the femoral head, rheumatoid arthritis, and hip dysplasia. In these cases, THA aims to restore or improve hip joint functionality, alleviate pain, and enhance patients’ quality of life^[1]^.

The assessment of physical function and joint performance before and after surgery necessitates the use of standardized outcome measures. However, the concept of “function” itself is complex and requires precise definition and measurement. Reiman *et al* addressed this issue by referring to the International Classification of Impairments, Disabilities, and Handicaps (ICF) disablement model, which conceptualizes function (or activity) as a highly intricate, multidimensional construct that varies among individuals^[6]^.

Various outcome measures can be used to assess physical function following THA. In both clinical practice and research, patient-reported outcome measures (PROMs) are the most commonly used tools to evaluate functionality and quality of life from the patient’s perspective. PROMs capture general health status, health-related quality of life (QoL), as well as pain and function of the affected hip joint. Commonly used PROMs include the Western Ontario and McMaster University Osteoarthritis Index (WOMAC), the Hip and Osteoarthritis Outcome Score (HOOS), and the Harris Hip Score (HHS). Despite their widespread use, PROMs provide only a subjective assessment by patients, which can be significantly influenced by psychological factors and pain perception. This may not be sufficient for a comprehensive evaluation of postoperative outcomes.

In contrast, objective measurements include performance-based outcome measures (PBOMs) and impairment-based outcome measures (IBOMs). Unlike PROMs, PBOMs and IBOMs do not rely on patients’ recall or self-assessment. PBOMs include tests such as the Timed Up and Go Test (TUG), the 6-Minute Walk Test, and the Sit-to-Stand Test (STS), which allow for an evaluation of hip joint functionality and provide insights into rehabilitation progress. PBOMs are more sensitive in detecting functional impairments compared to PROMs, as they assess functional abilities rather than subjective perceptions. However, they do not provide information on movement biomechanics or the underlying causes of deficits. IBOMs, such as gait analysis, range of motion (ROM) assessments, and muscle strength measurements, focus on specific aspects of hip function^[7–10]^.

The literature consistently emphasizes the importance of assessing function following THA. However, there is no consensus on which outcome measures should be used. This lack of agreement has led to considerable heterogeneity among clinical total joint replacement (TJR) studies. Standardized outcome measures are essential for valid comparisons between studies and for pooling data in meta-analyses. The Outcome Measures in Rheumatology and Clinical Trials (OMERACT) TJR working group, founded in 2008, includes international experts such as orthopedic surgeons, rheumatologists, physiotherapists, methodologists, and patient representatives. Systematic reviews revealed that inconsistent outcome measures limited comparability across TJR studies. Consequently, in 2014, the group established a core outcome set encompassing pain, function, patient satisfaction, revision surgeries, adverse events, and mortality^[5,11]^.

This study investigates how the domain of function has been assessed in THA research over the past 12 years and what insights can be derived from these assessments. This study hypothesizes that substantial variability exists among studies regarding the methodologies employed to assess physical function following THA.

## Methods

For this systematic review, the guidelines of the Preferred Reporting Items for Systematic Reviews and Meta-Analyses (PRISMA) were followed^[12,13]^, which represent the gold standard for transparent reporting of systematic reviews and meta-analyses^[14,15]^. Additionally, the methodological quality of the review process was assessed using the AMSTAR 2 (assessing the methodological quality of systematic reviews) tool^[16]^, a critical appraisal instrument for systematic reviews that include randomized or non-randomized studies of healthcare interventions. In addition, the TITAN guideline^[17]^, which provides recommendations for the responsible use of artificial intelligence in academic writing, was also followed and appropriately addressed. Compliance with these established guidelines ensures both rigorous methodology and transparent reporting. This study has been registered in PROSPERO under registration number: CRD42025643588.

### Search strategy

A comprehensive literature search was conducted using the electronic databases medline via PubMed, Ovid (Medline), ScienceDirect (Elsevier), and Epistemonikos (Embase, Cochrane Library, CINAHL, ClinicalTrials.gov, WHO ICTRP, OpenGrey and Web of Science) with the title keyword “total hip*.” The search encompassed studies published between January 2013 and February 2025, with a specific focus on randomized controlled trials (RCTs) that evaluated physical function, pain, and quality of life following THA. Both Medical Subject Headings (MeSH) and free-text terms were employed to ensure a thorough and exhaustive search. Language restrictions were applied to include only studies published in English and German. For example, the PubMed search string was:

(“total hip*”[Title]) AND((randomizedcontrolledtrial[Filter]) AND (2013:2025[pdat])).

**HIGHLIGHTS**

- Significant heterogeneity in outcome measures was observed across 406 randomized controlled trials (RCTs) assessing physical function post total hip arthroplasty (THA), with 65 distinct measurement tools identified.
- Patient-reported outcome measures (PROMs) dominated the literature (95% of studies), with the Harris Hip Score (HHS) being the most frequently used (35%), while performance-based (PBOMs, 18%) and impairment-based (IBOMs, 22%) measures were underutilized.
- Only 8% of studies integrated all three assessment types (PROMs, PBOMs, IBOMs), highlighting a gap in comprehensive functional evaluation despite recommendations from the OMERACT working group.
- Temporal trends revealed stable PROM usage (98–100% of studies annually), declining PBOM adoption (peaked at 30% in 2013–2014, then stabilized at 8–15%), and inconsistent IBOM application (11–36% annually).
- Critical limitations of PROMs were noted, including subjectivity influenced by pain perception and psychological factors, underscoring the need for complementary objective measures (PBOMs/IBOMs) to enhance validity.
- The lack of standardized outcome sets complicates cross-study comparisons and meta-analyses, emphasizing the urgency for core outcome sets to improve consistency in THA research and clinical practice.
- Rehabilitation-focused studies more frequently employed PBOMs and IBOMs (38% and 35%, respectively) than surgical studies (14% and 19%), reflecting their utility in assessing functional recovery.
- Key recommendation: Future studies should adopt multimodal assessment strategies (PROMs + PBOMs + IBOMs) aligned with OMERACT guidelines to ensure holistic evaluation of postoperative function and optimize patient outcomes

### Study selection

All records identified through the database searches were imported into EndNote (v.20, Clarivate Analytics, Philadelphia, PA, USA), and duplicate entries were removed. Two independent reviewers subsequently screened the titles and abstracts based on the predefined inclusion criteria. Full-text articles of potentially eligible studies were retrieved and assessed by the same reviewers. During full-text evaluation, reviewers also verified whether studies reported their funding sources; if this information was absent, it was documented as unreported. Any discrepancies arising during the selection process were resolved through discussion and, if necessary, by reaching a consensus with a third reviewer. The full texts were rigorously evaluated against the inclusion criteria detailed in Table 1, and studies that did not meet these criteria were excluded. Specifically, studies were excluded if they focused solely on radiological outcomes, addressed technical or surgical aspects without assessing physical function, or evaluated interventions unrelated to functional outcomes (e.g., assessments of anesthesia techniques or medication regimens).

**Table 1:** Presentation of the PICOS statement

Population	Adults who had undergone THA for any indication (e.g., osteoarthritis, trauma, degenerative joint disease)
Intervention/Exposure	Observational assessment of physical function post-THA using various outcome measurement tools
Comparison	Evaluation of different outcome measurement tools (PROMs, PBOMs, IBOMs) across studies
Outcome	Variability and frequency of use of different functional assessment tools
Study Design	Randomized controlled trials published between January 2013 and February 2025

### Data extraction

Data extraction was performed independently by two reviewers using a standardized extraction form. All data were manually collected in Microsoft Excel (without automated systems). In cases of disagreement during the extraction process, differences were addressed through discussion, with a third reviewer consulted to reach final consensus when needed. The extracted information was recorded in separate Excel spreadsheets and tabulated to include study title, authors, publication year, type of outcome measurements employed (i.e., PROMs, PBOMs, and IBOMs), the specific measurement tools used (e.g., HHS, WOMAC, TUG Test), and the core theme of each study. Studies were subsequently classified into three categories based on their primary focus: (a) surgery-related studies (e.g., material comparisons, surgical approach, instrumentation, robotic assistance, etc.), (b) post-operative rehabilitation studies, and (c) studies addressing other topics. For the purposes of this review, only outcome measurements pertaining to the domains of function and pain were considered. Any missing or unclear data were addressed by contacting the corresponding authors.

### Data synthesis

Data synthesis was performed using descriptive statistics. Studies were classified according to the types of outcome measurements employed: PROMs, which assess self-reported physical function, pain, and quality of life (e.g., HHS, WOMAC); PBOMs, which involve objective tests such as the TUG Test and Stair-Climbing Test; and IBOMs, which focus on specific impairments such as ROM or gait analysis (GA). The frequency and distribution of each measurement type and the specific tools used were calculated and presented in both tabular and graphical formats to illustrate trends over the study period. As the primary objective was to summarize the types and frequencies of functional assessment tools rather than to compare effect sizes or interventions, a meta-analysis was not conducted. All data were processed and analyzed using Microsoft Excel, and observed patterns and gaps in the use of outcome measures were discussed in the context of their implications for standardization in clinical practice.

## Results

### Results of study selection

**Figure 1.**
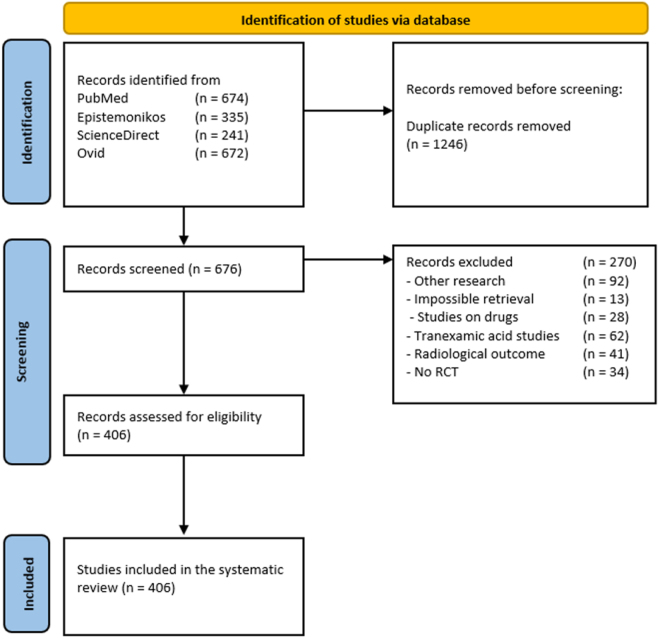
PRISMA flow chart.

A total of 1922 studies were initially identified based on the predefined search criteria. Following the initial screening, 1246 duplicate records were removed, leaving 676 unique records for further review. These records underwent a dual screening process conducted by two reviewers, during which 270 records were excluded for the following reasons: studies classified as other research (*n* = 92; e.g., cadaveric biomechanical analyses, animal model investigations, in vitro tissue assays), irretrievable full texts (*n* = 13), studies on drugs (*n* = 28; e.g., corticosteroid injection trials, non-antifibrinolytic hemostatic agent evaluations, novel analgesic drug interventions), tranexamic acid studies (*n* = 62; e.g., intra-articular TXA administration protocols, systemic antifibrinolytic regimens, topical TXA application assessments), studies reporting solely radiological outcomes (*n* = 41; e.g., CT-based implant alignment metrics), and studies that were not randomized controlled trials (*n* = 34; e.g., retrospective cohort studies, case-control studies). Consequently, 406 records remained for comprehensive examination. No additional exclusions were applied during subsequent screening stages, resulting in the final inclusion of 406 studies. The full list of these 406 references is provided in the supplementary Digital Content Materials, available at: http://links.lww.com/JS9/E933. A detailed flowchart of the study selection process is depicted in Fig. 1.

### Outcome measurements (PROMs, PBOMs, IBOMs)

**Figure 2.**
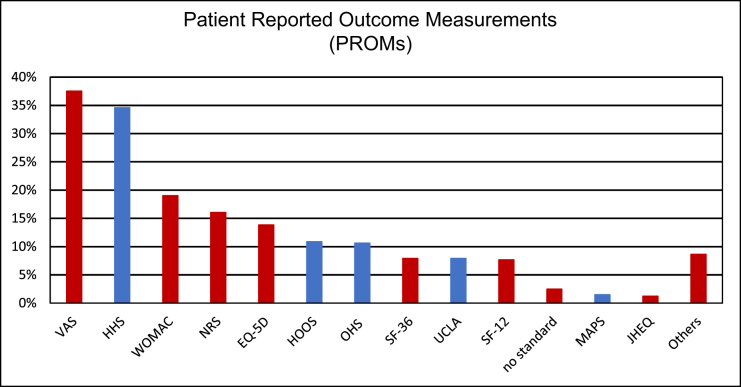
Detailed distribution of PROMs.

In the included studies, a total of 32 different PROMs, 26 PBOMs, and seven IBOMs were utilized to assess functional outcomes following THA. Overall, 384 studies (95%) employed at least one PROM. Among these, the most frequently employed instrument for assessing function (blue) were the HHS in 140 studies (35%), the HOOS in 44 studies (11%) and the OHS in 43 studies (11%). Furthermore, to evaluate pain and quality of life (QoL) domains (red), the most commonly used measurement tool was the VAS, applied in 152 studies (38%), followed by the WOMAC in 77 studies (19%), the NRS in 65 studies (16%) and the European Quality of Life 5 Dimensions (EQ-5D) in 56 studies (14%). Detailed results are presented in Figure 2.

The *y*-axes shows the percentage of studies that use the following PROMs**; VAS** (Visual Analog Scale), **HHS** (Harris Hip Score), **WOMAC** (Western Ontario McMaster Universities Index)**, NRS** (Numerical Rating Scale), **EQ-5D** (EuroQol-5D), **HOOS** (Hip disability and osteoarthritis outcome score), **OHS** (Oxford Hip Score), **SF-12**/**-36** (Short Form-12/-36), **UCLA** (University of California Los Angeles activity score), **MAPS** (Mayo Arthroplasty Performance Score**), JHEQ** (Japanese Hip Evaluation Questionnaire), **Others** (MDP (Merle d’Aubigné and Postel scores), LEFS (Lower Extremity Functional Scale), RAND-12/-36 (RAND-12/-36 Item Health Survey), GRC (Global Rating of Change scale), SANE (Single Assessment Numeric Evaluation), LASA (Linear Analogue Scale), FJS (Forgotten Joint Score), Charnley Score, NEADL (Nottingham Extended Activities of Daily Living Scale), FIM (Functional Independence Measure), SAPS (Self-Administered Patient Satisfaction Scale), IPAQ (International Physical Activity Questionnaire), PASE (Physical Activity Scale for the Elderly), HSQ (Health Screening Questionnaire), Staffelstein Score, XSFMA (extra short musculoskeletal functional assessment questionnaire), PAM (Patient Activation Measure), TSK (Tampa Scale of Kinesiophobia), MPQ-SF (McGill Pain Questionnaire Short Form), BPI-SF (Brief Pain Inventory-Short Form), SMH (St. Michales Hip Score).

**Figure 3.**
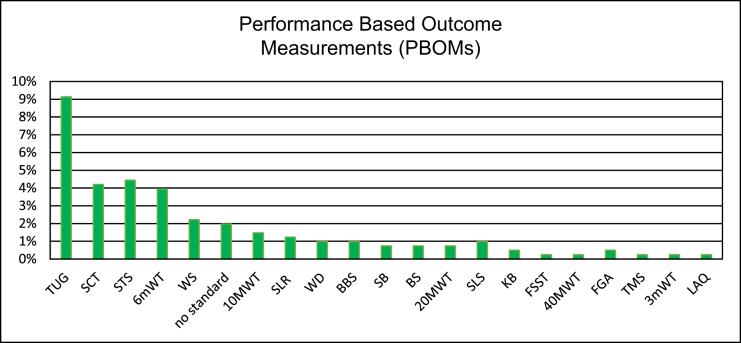
Detailed distribution of PBOMs.

To assess hip joint performance following THA, 73 studies (18%) employed at least one PBOM. Among these, the TUG test was the most frequently utilized, appearing in 37 studies (9%), followed by the Stair Climbing Test (SCT) in 17 studies (4%), Sit to Stand (STS) which was utilized in 18 studies (4%), and the 6-Minute Walk Test (6mWT) in 18 studies (5%). Detailed results are presented in Fig. 3.

The *y*-axes shows the percentage of studies that use the following PBOMs; **TUG** (Timed Up and Go), **SCT** (Stair Climbing-Test), **6mWT** (6-minute-Walk-Test), **WS** (Walking Speed), **STS** (Sit To Stand**), 4/10/20-MWT** (4/10/20-Meter-Walking Test), **SLS/DLS** (single/double leg stance), **TT** (Trendelenburg Test), **BI** (Barthel Index), **Activity Tracker, SLKB** (single-leg knee bending), **30s-CST** (30 seconds Chair-Stand-Test)**, SLR** (straight leg raise test), **BS** (Bromage Scale), **TMS** (Tinetti Mobility Scale), **SPPB** (Short Physical Performance Battery), **FSST** (four step in square test), **T25-FW** (timed 25-meter-foot-walk), **WBA** (Weight Bearing Amount), **WBR** (Wieght Bearing Ratio, **FR** (Functional Reach), **LAQ** (long arc quadriceps test), **3mWT** (3-minute-Walk-Test)

**Figure 4.**
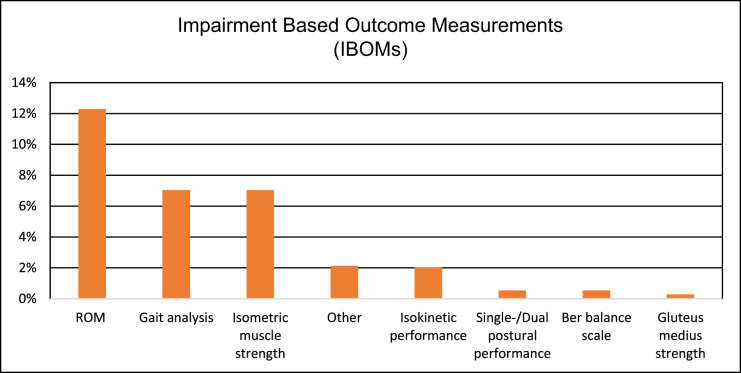
Detailed distribution of IBOMs.

Of the seven IBOMs utilized, at least one was employed in 90 studies (22%). ROM was the most frequently used IBOM, appearing in 40 studies (12%), followed by gait analysis in 27 studies (7%) and the measurement of isometric muscle strength in 26 studies (7%). A detailed analysis is presented in Fig. 4.

The *y*-axes show the percentage of studies that use IBOMs.

### Categorization by main topic

Of the 406 studies included, 195 (48%) were categorized as surgery-related studies. This category encompassed all studies investigating aspects such as surgical approach, material comparisons, instrumentation, or robotic assistance. Additionally, 117 studies (29%) focused on postoperative rehabilitation following THA, examining factors such as the effects of exercise, medication, and physiotherapy. The remaining 94 studies (23%) addressed other topics.

Among the three categories, surgery-related studies most frequently employed PROMs, with 189 studies (97%) utilizing at least one. In contrast, PBOMs (27 studies, 14%) and IBOMs (36 studies, 19%) were used less frequently. Studies focusing on postoperative rehabilitation incorporated PBOMs and IBOMs more often than surgery-related studies, highlighting their relevance in assessing functional recovery. In Table 2, the results are presented in more detail.

**Table 2:** Comparison of main topics and outcome measures

	Included studies (%)	Studies utilizing PROMs (%)	Studies utilizing PBOMs (%)	Studies utilizing IBOMs (%)
Surgery-related studies	195 (48)	189 (97)	27 (14)	36 (19)
Postoperative rehabilitation studies	117 (29)	104 (89)	38 (33)	41 (35)
Other areas	94 (23)	91 (97)	9 (10)	22 (24)
Total	406 (100)	384 (95)	74 (18)	99 (24)

### Combinations of PROMs, PBOMs, and IBOMs

**Figure 5.**
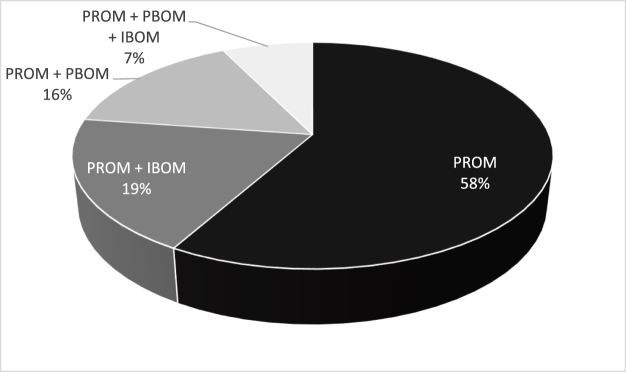
Combinations of PROMs, PBOMs, and IBOMs.

Several studies employed more than one type of outcome measure (PROM, PBOM, IBOM), such as a combination of a PROM with a PBOM or IBOM. A total of 236 studies (58%) utilized only PROMs to assess function. In contrast, 85 studies (19%) incorporated at least one PROM and one IBOM. Furthermore, 69 studies (16%) applied at least one PROM and one PBOM, while 32 studies (7%) utilized all three types of outcome measures for the comprehensive assessment of function as presented in Figure 5.

### Temporal trends in the use of PROMs, PBOMs, and IBOMs

The utilization of PROMs has remained consistently high over the past 12 years, with a prevalence ranging from 98% to 100% in the studies examined. In contrast, PBOMs were used in approximately 30% of studies in 2013 and 2014 but declined in frequency over the years (Pearson correlation [*r* = −0.69]). Despite the decrease of PBOM usage, inconstancy can be observed over the years.

**Figure 6.**
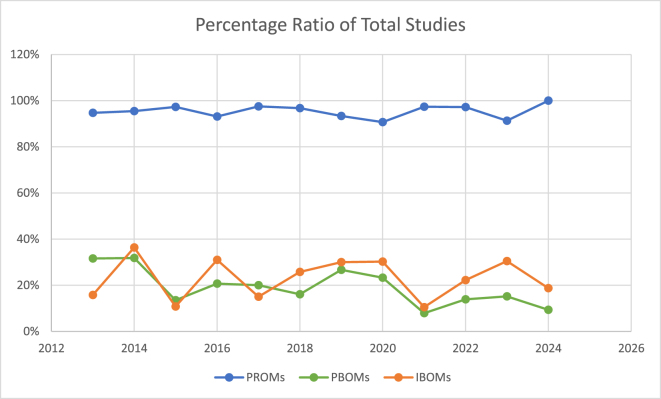
Presentation of the utilization of PROMs, PBOMs, and IBOMs over the years.

The application of IBOMs does not exhibit a clear trend toward increased adoption in the reviewed studies. Their utilization has fluctuated between 36% in 2014 and 11% in 2021, indicating no consistent pattern of growth in their application. The detailed trends over the investigated years are presented in Figure 6.

## Discussion

The most important findings of this review reveal substantial variability in how physical function is assessed following THA. Our analysis of 406 studies published over the past 12 years identified 65 different measurement methods, with PROMs being the most frequently used—employed exclusively in 58% of studies. This preference for PROMs likely stems from their standardized nature, ease of administration, and ability to be completed independently by patients. However, the striking lack of consensus is evident in the fact that no single measurement method was used in more than half of the studies.

Analysis of temporal trends shows that PROMs have remained almost universally adopted (98–100% prevalence) over the past 12 years, whereas PBOM usage fell from about 30% in 2013–2014 to a low of 8–15% after 2019, despite a brief resurgence to 27% in 2019. This decline may reflect increasing reliance on remote or digital data collection, resource and staffing constraints for in-person performance tests, and the impact of the COVID-19 pandemic on clinical assessments. In contrast, IBOM utilization has fluctuated without a clear upward trajectory (36% in 2014 down to 11% in 2021), underscoring the lack of sustained growth in more specialized physiological measures.

A comprehensive understanding of physical function requires the complementary use of different assessment approaches. PBOMs provide objective data through standardized tests of mobility, strength and endurance, while IBOMs quantify specific physiological limitations like muscle weakness or restricted joint mobility. In contrast, PROMs offer invaluable insights into patients’ subjective experiences of function and pain, capturing aspects that may not be apparent through clinical measures alone (Table 3).

**Table 3:** Summary of predominantly used PROMs, PBOMs and IBOMs

	PROMs	PBOMs	IBOMs
Focus	Subjective perception of function, pain, and quality of life	Functional performance and capability	Physiological or structural impairments
Assessment method	Questionnaires, rating scales, or interviews	Standardized tests involving real tasks	Biomechanical or physiological measurements
Outcome representation	Scores based on patient responses	Time, distance, repetitions, speed, etc.	Muscle strength, ROM, pain threshold
Example assessments	HHS, WOMAC, HOOS, OHS, SF-36, EQ-5D	TUG, 6MWT, STS	Isometric strength testing, ROM, EMG
Objectivity	Low to moderate (subjective patient- reported responses)	High (standardized conditions)	High (quantifiable physiological data)
Relevance to daily life	High (reflects patient- perceived limitations and quality of life)	High (demonstrates actual performance ability)	Low to moderate (identifies impairments but not necessarily functional impact)
Advantages	Captures the patient’s personal perception of symptoms and limitations	Direct measurement of functional capacity	Identifies specific physiological deficits
Disadvantages	Subjective, potentially influenced by expectations or emotional state	Performance may be influenced by motivation and daily variability	May lack real-world applicability

This heterogeneity in assessment tools, particularly the wide variation in PROM selection and application, presents significant challenges for evidence synthesis. The lack of standardized measures complicates systematic reviews and meta-analyses^[3]^, potentially limiting our ability to draw robust conclusions across studies.

he marked decline in PBOM adoption post-2019 warrants deeper consideration. Practical barriers such as limited appointment times, lack of dedicated testing equipment, and increasing pressures on allied health staff have likely played a central role^[18]^. Within the UK’s NHS context, well-documented shortages in physiotherapy staffing and a shift towards telehealth consultations have constrained opportunities for supervised performance testing^[19]^. Furthermore, ongoing debates about the validity and interpretability of certain PBOMs may have discouraged their routine use, as clinicians and researchers grapple with inconsistent normative data and variable inter-rater reliability^[20]^.

Despite growing calls for standardization, the barriers to widespread PBOM/IBOM adoption remain under-explored. NICE Quality Standard 206 notes that delivering postoperative rehabilitation, including early mobilization, often necessitates additional physiotherapy cover at weekends, imposing significant staffing and financial demands on NHS trust^[21]^. Moreover, NICE Guideline 157 highlights marked variability in rehabilitation service provision and indicates that many NHS clinicians lack the specialist training and equipment required to perform standardized PBOMs such as gait analysis or instrumented strength testing^[22]^. These cost and training constraints must be addressed when developing Core Outcome Sets (COS) to ensure their feasibility and sustainability within resource-limited healthcare settings.

Furthermore, a study by Dayton *et al* demonstrated that PROMs alone are insufficient to comprehensively evaluate outcomes following THA, as patients tend to overestimate their functional performance. Therefore, the authors recommend incorporating both PROMs and PBOMs to assess outcomes more accurately^[23]^. In this review, only 17% of the identified studies employed a combination of PROMs and PBOMs to evaluate function.

As highlighted in the introduction, assessing physical function remains a significant challenge. Notably, the authors of the included studies did not always explicitly prioritize physical function as the primary focus of their research. Nonetheless, PROMs, PBOMs, and IBOMs collectively aim to capture different aspects of functional performance.

Although all these outcome measures contribute valuable insights, they each have inherent limitations. PROMs are based on patient self-assessment, which is significantly influenced by pain perception. For example, while patients undergoing THA reported significant improvements in their perceived functional abilities following pain reduction, they still required twice as much time to complete functional tasks. This suggests that patients may overestimate their functional capacity when experiencing less pain. Therefore, while PROMs serve as an essential tool for evaluating physical function, they should be interpreted with and considered as only one component of functional assessment^[6]^. Additionally, the subjective nature of PROMs makes them susceptible to psychological factors such as anxiety or depression, which may distort the assessment of physical function. Furthermore, as postoperative PROMs are strongly influenced by a patient’s current pain status, they may not accurately reflect actual functional performance.

PBOMs, considered functional performance tests, typically measure only a single functional parameter and should therefore be regarded primarily as assessments of physical capacity. Although they are frequently utilized in rehabilitation studies, they do not necessarily predict successful functional recovery or future injuries. This assertion is supported by findings from the present review: studies focusing on postoperative rehabilitation more commonly employ PBOMs compared to those with a surgical approach. Additionally, PBOMs represent only a snapshot of performance, which can be influenced by factors such as pain, well-being, and overall physical condition^[23,24]^.

IBOMs require more time to administer compared to PROMs and PBOMs. According to Reiman *et al*, impairments refer to dysfunctions or structural abnormalities in a specific body part or system, potentially leading to functional limitations and ultimately affecting physical performance. Clinical special tests are used to identify pain sources but exhibit deficiencies in sensitivity and specificity. Consequently, the presence of an impairment on clinical examination does not necessarily equate to functional loss^[6,9]^.

Furthermore, it is noteworthy that while PROMs have been consistently and reliably utilized over the past 12 years, this has not been the case for PBOMs and IBOMs, which have been applied less consistently.

Pure pain and QoL outcome measures (VAS, EQ-5D-5L, NRS) were utilized only 282 times across the included studies. Notably, this count does not account for subscales, such as those from the WOMAC, that assess pain. These subscales frequently integrate questions related to both function and pain. Stratford *et al* investigated the limited ability of WOMAC subscales to distinguish between pain and functional changes. Their findings revealed that overlapping questions in the pain and function subscales impair the measure’s ability to detect changes^[24]^.

The relative paucity of long-term functional assessment data, beyond 1 year post-THA, also warrants deeper consideration. Without robust longitudinal outcomes, clinicians lack evidence on sustained recovery trajectories, potentially biasing decisions toward interventions demonstrating only short-term gains. In the absence of extended follow-up, recommendations for timing of rehabilitation milestones and patient counseling on realistic long-term expectations remain speculative. Future investigations must prioritize multi-year follow-up of PROMs, PBOMs, and IBOMs to inform clinical guidelines and optimize long-term patient outcomes.

Of the 406 studies analyzed, only 30 (8%) incorporated a combination of all three outcome measures (PROMs, PBOMs, and IBOMs), representing the most comprehensive approach to assessing function and aligning with the recommendations of the OMERACT working group. A common criticism of PROMs is that they may not accurately reflect actual functional capacity as measured by PBOMs or IBOMs^[25–27]^. This underscores the importance of integrating multiple assessment methods to provide a more complete evaluation of patient function. Implementing a standardized set of outcome measures could enhance the comparability of study findings, facilitate the development of more effective rehabilitation strategies, and ultimately improve patient outcomes^[5,11,28]^.

Although OMERACT has advocated for a COS to harmonize assessment across THA trials, practical guidance on its implementation remains lacking. An actionable COS might combine a PROM such as the HHS, a PBOM like the TUG test, and an IBOM such as instrumented gait analysis. Yet within the NHS, time pressures, constrained physiotherapy staffing levels, and limited access to gait laboratories raise concerns regarding feasibility and cost-effectiveness. Early adopters suggest that integrating COS into routine care will require investment in digital PROM platforms, dedicated personnel for performance testing, and strategic use of existing community physiotherapy services to conduct PBOMs. Addressing these logistical challenges is essential to ensure that a standardized COS can be both scientifically robust and operationally sustainable across the NHS.

### Limitations and strengths

This systematic review has several strengths and limitations that must be considered. One of its key strengths is the inclusion of only RCTs published within the last 12 years, ensuring a high methodological standard. However, this approach also introduces a limitation, as it excludes prospective observational studies and cross-sectional clinical studies that report on functional outcome measurements. Consequently, while the included studies provide robust methodological quality, this review may not offer a fully comprehensive overview of the outcome measures currently used in the literature.

Furthermore, by restricting our sample to RCTs, we may have inadvertently overestimated the prevalence of PROMs. Randomized trials frequently prioritize patient-reported endpoints to satisfy regulatory requirements, whereas observational cohorts often employ a broader mix of objective performance tests. This exclusion thus limits the generalizability of our findings to real-world practice, where PBOMs and IBOMs might be more common.

While this systematic review did not formally assess individual studies for levels of risk of bias (e.g., low, moderate, or high), publication bias, or methodological quality, this approach was methodologically justified by the review’s primary objective. As the focus centered on cataloging and analyzing the types of PROMs, PBOMs, and IBOMs employed across studies, rather than evaluating intervention efficacy, traditional quality assessment metrics were deemed less critical to the research question. The selection and reporting of outcome measures exists largely independently of study quality parameters, making formal bias assessment non-essential for addressing the specific aims. This approach aligns with similar methodological reviews prioritizing instrument characterization over therapeutic effectiveness evaluation. Nonetheless, omitting a structured risk-of-bias assessment (e.g., Cochrane RoB 2.0) diminishes methodological rigor, as it may fail to detect biases, such as a tendency for industry-funded trials to preferentially employ PROMs, and thus potentially skew the characterization of outcome measures and the review’s conclusions.

Additionally, a considerable challenge in synthesizing the available evidence is the heterogeneity of outcome measures. The wide variety of PROMs, PBOMs, and IBOMs complicates direct comparisons across studies, making it impossible to conduct a meta-analysis.

A further limitation is the lack of long-term data. Many included studies primarily focus on short-term postoperative outcomes (e.g., within 1 year), whereas data on longer-term functional outcomes following THA remain scarce. This gap in the literature limits the ability to assess the sustained effectiveness of different interventions over time.

Moreover, the search strategy was restricted to English- and German-language publications, potentially overlooking relevant studies published in other languages. In particular, studies indexed in the China National Knowledge Infrastructure database were not included, despite the fact that China has contributed a substantial number of high-quality RCTs on THA, albeit primarily in Chinese.

Finally, the review may have underestimated the role of psychosocial factors, comorbidities, and ethnic or cultural differences in functional outcomes.

## Conclusion

This review highlights considerable variability in outcome measures, with PBOMs and IBOMs being significantly underutilized compared to PROMs. Only a small proportion of studies incorporate all three assessment types, and no clear trend toward greater use of PBOMs or IBOMs has emerged over the past 12 years. To enhance comparability across studies, improve the evaluation of functional recovery, and optimize patient rehabilitation and outcome, future research should implement standardized core outcome sets, such as those recommended by OMERACT. This would enable more consistent reporting and facilitate a more comprehensive assessment of patient outcomes and rehabilitation strategies.

## Data Availability

The datasets generated and/or analyzed during this study are available from the corresponding author upon reasonable request.

## Research registration unique identifying number (UIN)

PROSPERO registration number: CRD42025643588.
